# Is urinary density an adequate predictor of urinary osmolality?

**DOI:** 10.1186/s12882-015-0038-0

**Published:** 2015-04-08

**Authors:** Ana Carolina P Souza, Roberto Zatz, Rodrigo B de Oliveira, Mirela A R Santinho, Marcia Ribalta, João E Romão, Rosilene M Elias

**Affiliations:** Nephrology Service, University of Sao Paulo School of Medicine, São Paulo, SP Brazil; Nephrology Service, University of Campinas - UNICAMP, Campinas, Brazil

**Keywords:** Kidney disease, Urinalysis, Urine density, Urine osmolality, Urine concentrating ability

## Abstract

**Background:**

Urinary density (UD) has been routinely used for decades as a surrogate marker for urine osmolality (U_osm_). We asked if UD can accurately estimate U_osm_ both in healthy subjects and in different clinical scenarios of kidney disease.

**Methods:**

UD was assessed by refractometry. U_osm_ was measured by freezing point depression in spot urines obtained from healthy volunteers (N = 97) and in 319 inpatients with acute kidney injury (N = 95), primary glomerulophaties (N = 118) or chronic kidney disease (N = 106).

**Results:**

UD and U_osm_ correlated in all groups (p < 0.05). However, a wide range of U_osm_ values was associated with each UD value. When UD was ≤ 1.010, 28.4% of samples had U_osm_ above 350 mOsm/kg. Conversely, in 61.6% of samples with UD above 1.020, U_osm_ was below 600 mOsm/kg. As expected, U_osm_ exhibited a strong relationship with serum creatinine (S_creat_), whereas a much weaker correlation was found between UD and S_creat_.

**Conclusion:**

We found that UD is not a substitute for U_osm_. Although UD was significantly correlated with U_osm_, the wide dispersion makes it impossible to use UD as a dependable clinical estimate of U_osm_. Evaluation of the renal concentrating ability should be based on direct determination of U_osm_.

## Background

Measurement of urine osmolality (U_osm_), the gold standard in the evaluation of urine concentrating ability, is a valuable tool for the assessment of renal function in such distinct clinical conditions as acute kidney injury (AKI) and chronic kidney disease (CKD). However, since U_osm_ is not routinely measured, assessment of urine density (UD) by hydrometry, refractometry or semi-quantitative colorimetric reactions has long been employed instead.

Although a correlation does exist between UD and U_osm_, at least under normal physiological conditions [1-4], the assumption that UD accurately reflects U_osm_, which underlies any clinical decision based on UD, has not been formally tested and, in fact, has been recently challenged [5,6].

In the present study we examined the relation between UD (measured by refractometry) and U_osm_ in 97 normal subjects as well as in a cohort of 319 patients with assorted renal disorders, to test the hypothesis that UD can be reliably used in routine clinical practice as a measure of U_osm_.

## Methods

Urine samples were consecutively obtained from 95 adult patients with AKI in intensive care unit, 118 patients with primary glomerulopathies, admitted to a Nephrology ward for investigation, 106 CKD outpatients, and 97 healthy volunteers. Urine samples were obtained from the first morning void, with no standardized water restriction. This study protocol was reviewed and approved by our Institutional Research Ethics Committee (Comissão de Ética para Análise de Projetos de Pesquisa, CAPPesq, #0045/08). A written informed consent was obtained.

UD was measured by refractometry, employing a benchtop refractometer (ATAGO CO.LTD SPR-T2, Tokyo, Japan). Results were corrected for the influence of protein and/or glucose according to conventional equations [6]: UD corrected for proteinuria = UD measured – [protein] *0.0003; UD corrected for glucosuria = UD measured – [glucose] *0.0002, where both protein and glucose in the urine are given in g/L. U_osm_ was measured by freezing point depression using an advanced wide-range osmometer (Model 3 W2, Advanced Instruments Inc, Needham Heights, Massachusetts). S_Creat_ was measured with a conventional automated method (enzymatic colorimetric test, by the Jaffe reaction).

Serum sodium concentration was obtained in 203 patients (63.3%). Hypernatremia was found in 3 patients (147 to 149 mEq/l), whereas hyponatremia was observed in 8 patients (129 to 134 mEq/l). Since dysnatremia was infrequent, and never severe, this data was not further analyzed.

AKI was defined according to the current KDIGO classification [7]. In nearly all patients with glomerulopathies, hospitalization was indicated for kidney biopsy and/or clinical management of nephritic or nephrotic syndrome.

### Statistical analysis

Univariate correlation analysis between single variables was performed by calculating the Spearman coefficient. Data management was performed by Prism 6.0 statistical software (Graphpad, San Diego, CA, USA). A two-tailed p value < 0.05 was considered statistically significant.

## Results

Spot urine samples from 97 subjects with normal renal function (Control Group) and from 319 inpatients with acute kidney injury (AKI, N = 95), glomerulopathies (GP, N = 118) or chronic kidney disease (CKD, N = 106) were analyzed.

**Figure 1:**
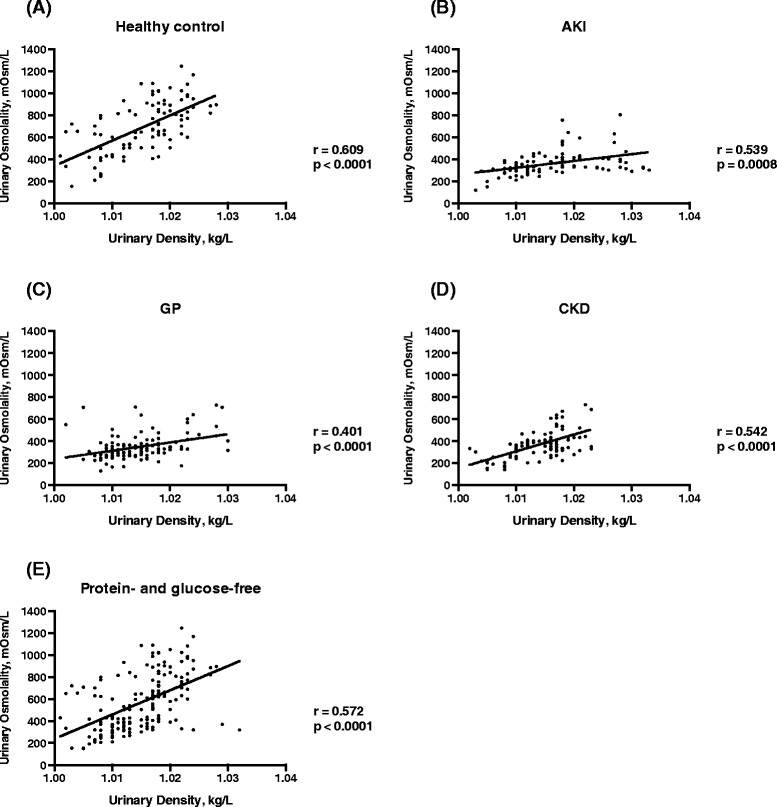
**Correlation between urinary osmolality (U**_**osm**_ **) and urinary density (UD) in each subgroup of patients: healthy control (A), acute kidney injury – AKI (B), glomerulopathies - GP (C), chronic kidney disease – CKD (D), and the entire group with urine free of protein and glucose (E).**

As expected, UD was consistently correlated to U_osm_. Correlation was statistically significant when all groups were considered together (r = 0.462, p < 0.0001), as well as in the healthy control group (r = 0.609, p < 0.0001); in the AKI group (r = 0.539, p = 0.0008); in the GP group (r = 0.401, p < 0.0001); and in the CKD group (r = 0.542, p < 0.0001), as shown in Figure 1A, B, C and D, respectively. UD was corrected for proteinuria and glucosuria as described under Methods. Proteinuria was found in almost 60% of samples: overall, only 171 out of 416 samples were free from proteinuria or glucosuria (all 98 healthy volunteers, 35 out of 95 in the AKI group, 35 out of 106 in the CKD group, and only 3 out of 118 in the GP group). Glucosuria was found in only 5 patients from the CKD group and in 4 patients from the GP group. These 9 samples also exhibited proteinuria, and therefore both equations were used for correction. When only urine samples without proteinuria or glucosuria were analyzed, the correlation between UD and U_osm_ was even stronger (r = 0.572, p < 0.0001) (Figure 1E).

**Figure 2:**
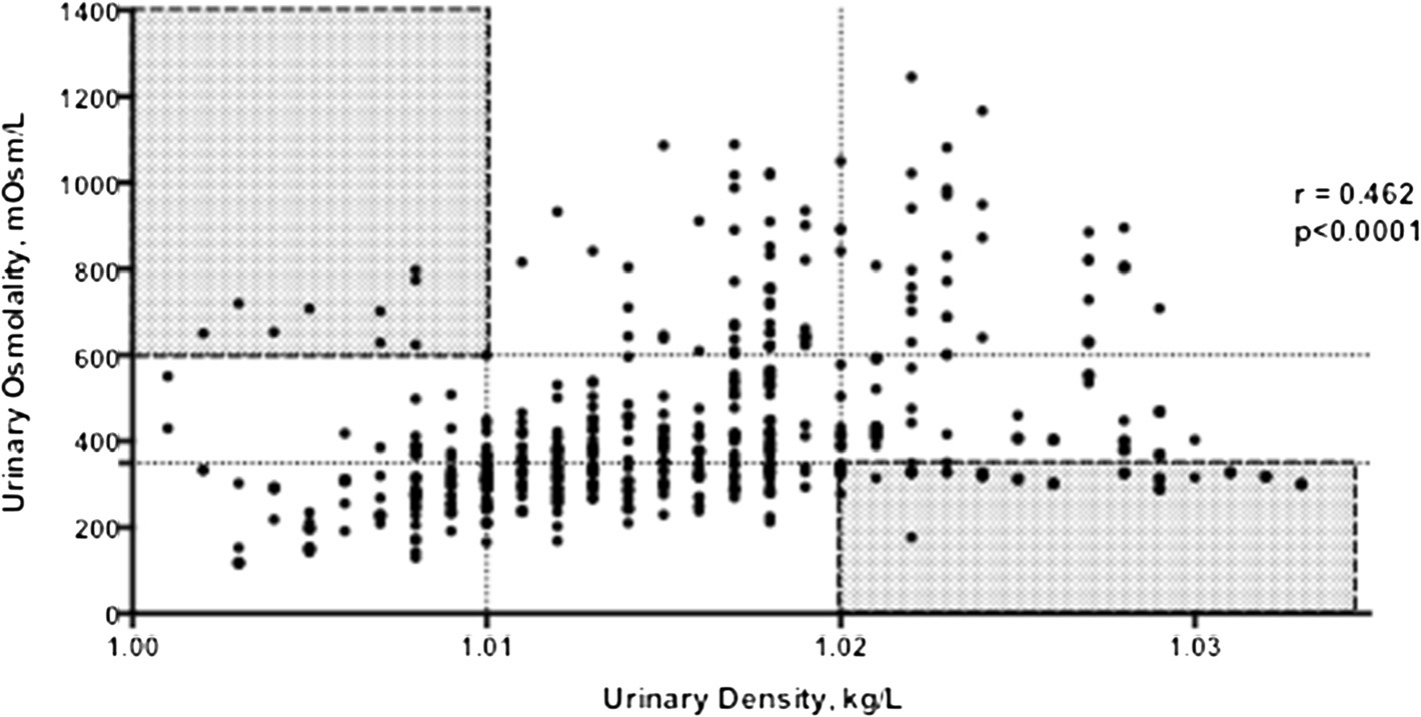
**Correlation between urinary osmolality (U**_**osm**_ **) and urinary density (UD) in all groups.** Shaded areas are pointing to unexpected high or low UD while U_osm_ was the opposite.

Despite the significant correlation between UD and U_osm_, a wide range of U_osm_ values was associated with each UD value. This inconsistency was particularly striking when extreme UD values were considered (Figure 2, shaded areas): 39.8% of samples with UD lower or equal to 1.010 exhibited U_osm_ in excess of 350 mOsm/kg. Conversely, in 58.6% of samples with UD above 1.020, U_osm_ was below 600 mOsm/kg.

Low, normal and high renal concentration ability was defined as UD lower or equal to 1.010, between 1.010 and 1.020, and above or equal to1.020 kg/L, and U_osm_ lower than 350 mOsm/kg, between 350 and 600 mOsm/kg, and higher than 600 mOsm/kg, respectively. The number of patients classified in each criterion is plotted in Table 1. Agreement between UD and U_osm_ is highlighted in gray.

**Table 1:** **Comparison between urinary density (UD) and osmolality (U**_**osm**_ **) classification of low and high concentration ability**

**UD criteria**	**Osmolality criteria**		
**All patients**	**Low (<350mOsm/kg)**	**Normal (350-600mOsm/kg)**	**High (>600mOsm/kg)**
Low UD (≤1.010 kg/L)	**71**	27	20
Normal UD (1.011 – 1.019 kg/L)	79	**89**	43
High (UD ≥1.020 kg/L)	26	25	**36**
**Patients with AKI**			
Low UD (≤1.010 kg/L)	**28**	2	0
Normal UD (1.011 – 1.019 kg/L)	20	**17**	2
High (UD ≥1.020 kg/L)	13	11	**2**
**Patients with CKD**			
Low UD (≤1.010 kg/L)	**8**	10	10
Normal UD (1.011 – 1.019 kg/L)	23	**38**	6
High (UD ≥1.020 kg/L)	3	5	**3**
**Patients with glomerulonephritis**			
Low UD (≤1.010 kg/L)	**28**	7	1
Normal UD (1.011 – 1.019 kg/L)	36	**21**	2
High (UD ≥1.020 kg/L)	10	9	**4**
**Healthy individuals**			
Low UD (≤1.010 kg/L)	**7**	8	9
Normal UD (1.011 – 1.019 kg/L)	0	**13**	33
High (UD ≥1.020 kg/L)	0	0	**27**

Bold numbers show agreement between UD and U_osm_.

AKI, acute kidney injury; CKD, chronic kidney disease.

When analyzing healthy subjects, UD was an excellent predictor of U_osm_ when it was above 1.020: 100% of these samples exhibited U_osm_ above 600 mOsm/kg. By sharp contrast, UD failed to predict U_osm_ when UD was below or equal to 1.010: only 29.2% of samples had U_osm_ lower than 350 mOsm/kg, while U_osm_ was higher than 600 mOsm/kg in 37.5% of the samples.

**Figure 3:**
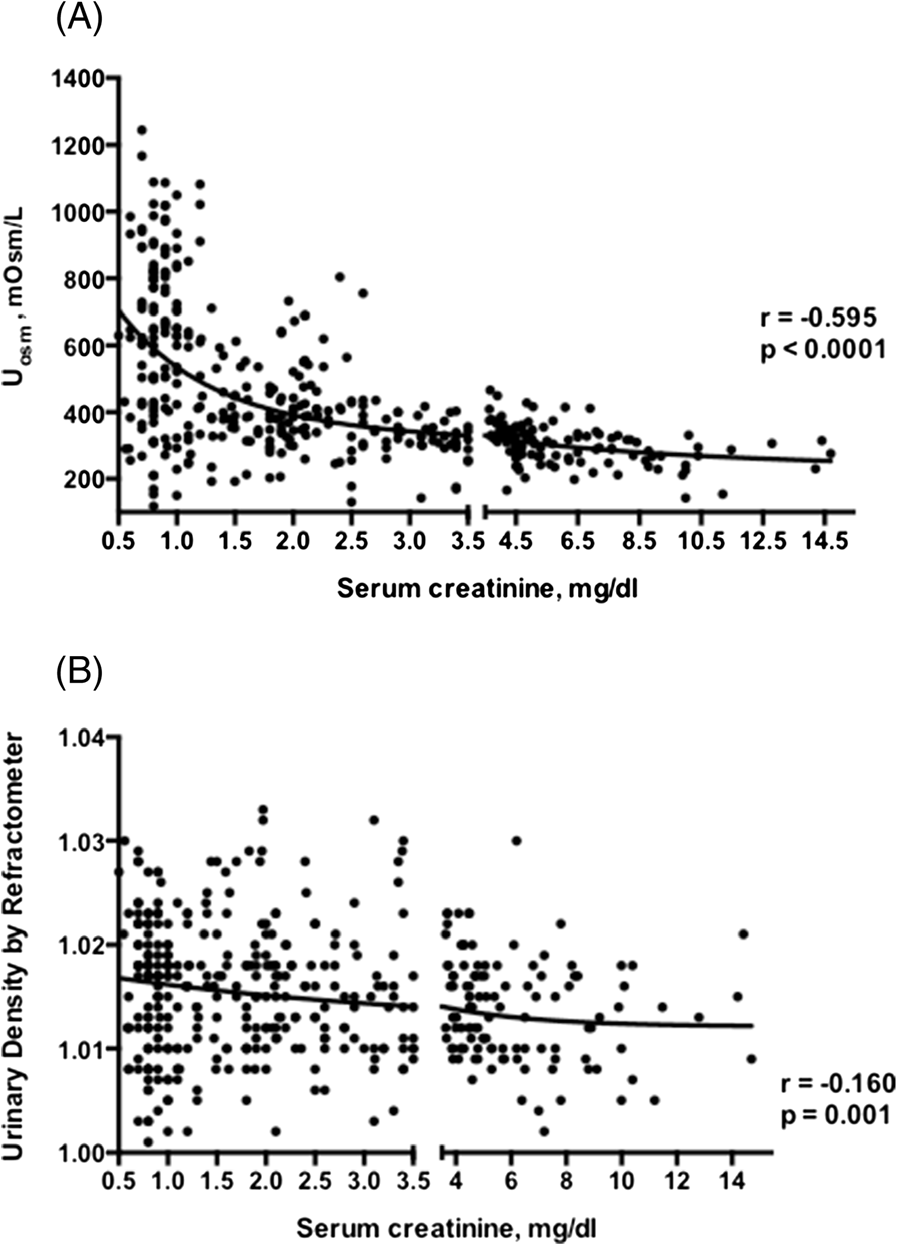
**Correlation between serum creatinine (S**_**Creat**_ **) levels and urinary osmolality (U**_**osm**_ **) (A), and urinary density (UD) (B).**

Figure 3A illustrates the relationship between U_osm_ and serum creatinine (S_creat_), for all subjects. U_osm_ and S_creat_ followed a nonlinear relationship that could be fitted to a two-exponential curve (p < 0.01): U_osm_ was expectedly distributed across a wide range (118–1245 mOsm/kg) in subjects with S_creat_ lower than 1.0 mg/dL and was confined to a narrow interval around 300 mOsm/kg as S_creat_ increased. The relationship between U_osm_ and S_creat_ was still significant (p < 0.05), although much less conspicuous, when UD replaced U_osm_ as a measure of urine concentration (Figure 3B). When analyzing subgroups, the correlation between U_osm_ and S_creat_ was significant in the AKI group (r = −0.451, p < 0.001), in the GP group (r = −0.533, p = 0.0001), and in the CKD group (r = −0.546, p = 0.0001). There was no significant correlation between U_osm_ and S_creat_ in the healthy control group (r = −0.108, p = 0.289). A weaker but still significant correlation between UD and S_creat_ was shown in the AKI group (r = −0.160, p = 0.001), in the GP group (r = −0.253, p = 0.026), and in the CKD group (r = −0.209, p = 0.031). There was no correlation between UD and S_creat_ in the healthy control group (r = −0.102, p = 0.863).

## Discussion

Urine density has long been considered as a practical surrogate marker of urine osmolality. It has even been proposed that simple equations be used in clinical practice to obtain U_osm_ directly from UD [8-11], whereas a website offers such calculations online [12]. In the present study, we challenged the concept that UD is a reliable marker of urine osmolality. For better accuracy, UD measurements were made utilizing a refractometer, instead of the semi-quantitative dipstick method more commonly employed. Even so, the correlation obtained between UD and U_osm_, though statistically significant, was relatively weak (r = 0.462). A closer examination casts serious doubts about the clinical usefulness of UD. If an UD of 1.020 kg/L or higher were regarded as a test to detect individuals with an U_osm_ of at least 600 mOsm/kg [8-10], the sensitivity of such a test would be only 36%, whereas its specificity would be 81%. In other words, 64% of the subjects with concentrated urines would be missed by such test. On the other hand, good renal concentration ability might be erroneously inferred in as many as 19% of the other cases. Conversely, if an UD equal to or less than 1.010 kg/L were assumed to detect urine osmolalities below 350 mOsm/kg (isosthenuric or diluted urine), the sensitivity of the test would be only 40%, although the corresponding specificity would approach a more acceptable 80%.

The inadequacy of UD as a measure of U_osm_ becomes even more evident when we consider that in about 2/3 of all samples UD values were ≥1.010 and ≤1.020 kg/L, thus being irrelevant to major clinical decisions such as whether or not to administer IV saline to hypovolemic patients. When only UDs equal or higher than 1.020 kg/L were analyzed, a mere 41.4% of the samples actually exhibited U_osm_ above 600 mOsm/kg, whereas in 29.9% U_osm_ was even below 350 mOsm/kg. In the converse case, when exclusively UDs equal or lower than 1.010 kg/L were considered, 60.2% of samples exhibited U_osm_ values lower than 350 mOsm/kg, whereas in 16.9% U_osm_ even exceeded 600 mOsm/kg. These results indicate that UD cannot replace U_osm_ as a faithful measurement of urine concentration. Although a high U_osm_ may not reflect the actual volume status, such as in cases of inappropriate antidiuretic hormone secretion, in many situations it does signal the need for fluid replacement. Therefore, an erroneous interpretation of a high UD as indicative of hypovolemia may lead to inadequate and even dangerous fluid administration.

As expected, there was a significant nonlinear correlation between U_osm_ and S_creat_, reflecting the expected relationship between renal function and urine concentrating/diluting ability: individuals with normal renal function can vary urine osmolality over a wide range, while renal functional impairment is accompanied by a progressive limitation of this capacity, until urine becomes permanently isosthenuric as most of the renal function is lost. This relationship between U_osm_ and S_creat_ was strongly attenuated when UD was used instead of U_osm_, reinforcing the view that UD is a poor marker of renal concentrating/diluting ability.

The reasons why the relationship between UD and U_osm_ is less consistent than might be expected are unclear. In the present study, the effect of the possible presence of glucose and/or protein in urine was corrected by applying appropriate equations [6,13]. However, the association between UD and U_osm_ remained loose even after samples containing these solutes were excluded (r = 0.459, p < 0.05). It should be noted that a myriad of other solutes, commonly encountered in the urine of patients with renal disorders, such as drugs and iodinated radiocontrast agents, could increase urine density, leading to overestimation of the renal concentrating ability. Even “physiologic” solutes, such as sodium, potassium and urea, can appear in widely varying proportions in the urine of both healthy and diseased subjects, each of them exerting a different influence on urine density [9]. The unpredictability of these effects helps to explain the erratic relationship between UD and U_osm_.

## Conclusion

In summary, although UD correlates with U_osm_, the relationship between these two parameters is largely inconsistent, even in healthy subjects, indicating that UD is a poor marker of renal concentrating/diluting capability. Direct determination of U_osm_, a relatively inexpensive procedure, should be performed if reliable information about this important aspect of renal function is to be obtained from urine.
